# Epigenetic regulation in adult neural stem cells

**DOI:** 10.3389/fcell.2024.1331074

**Published:** 2024-01-31

**Authors:** Jiajia Shi, Zilin Wang, Zhijun Wang, Guofeng Shao, Xiajun Li

**Affiliations:** ^1^ School of Life Science and Technology, ShanghaiTech University, Shanghai, China; ^2^ Zhenhai Lianhua Hospital, Ningbo City, Zhejiang, China; ^3^ Department of Cardiothoracic Surgery, Lihuili Hospital Affiliated to Ningbo University, Ningbo City, Zhejiang, China

**Keywords:** neural stem cell (NSC), epigenetic, transcription, adult brain, DNA methylation

## Abstract

Neural stem cells (NSCs) exhibit self-renewing and multipotential properties. Adult NSCs are located in two neurogenic regions of adult brain: the ventricular-subventricular zone (V-SVZ) of the lateral ventricle and the subgranular zone of the dentate gyrus in the hippocampus. Maintenance and differentiation of adult NSCs are regulated by both intrinsic and extrinsic signals that may be integrated through expression of some key factors in the adult NSCs. A number of transcription factors have been shown to play essential roles in transcriptional regulation of NSC cell fate transitions in the adult brain. Epigenetic regulators have also emerged as key players in regulation of NSCs, neural progenitor cells and their differentiated progeny via epigenetic modifications including DNA methylation, histone modifications, chromatin remodeling and RNA-mediated transcriptional regulation. This minireview is primarily focused on epigenetic regulations of adult NSCs during adult neurogenesis, in conjunction with transcriptional regulation in these processes.

## Introduction

**FIGURE 1:**
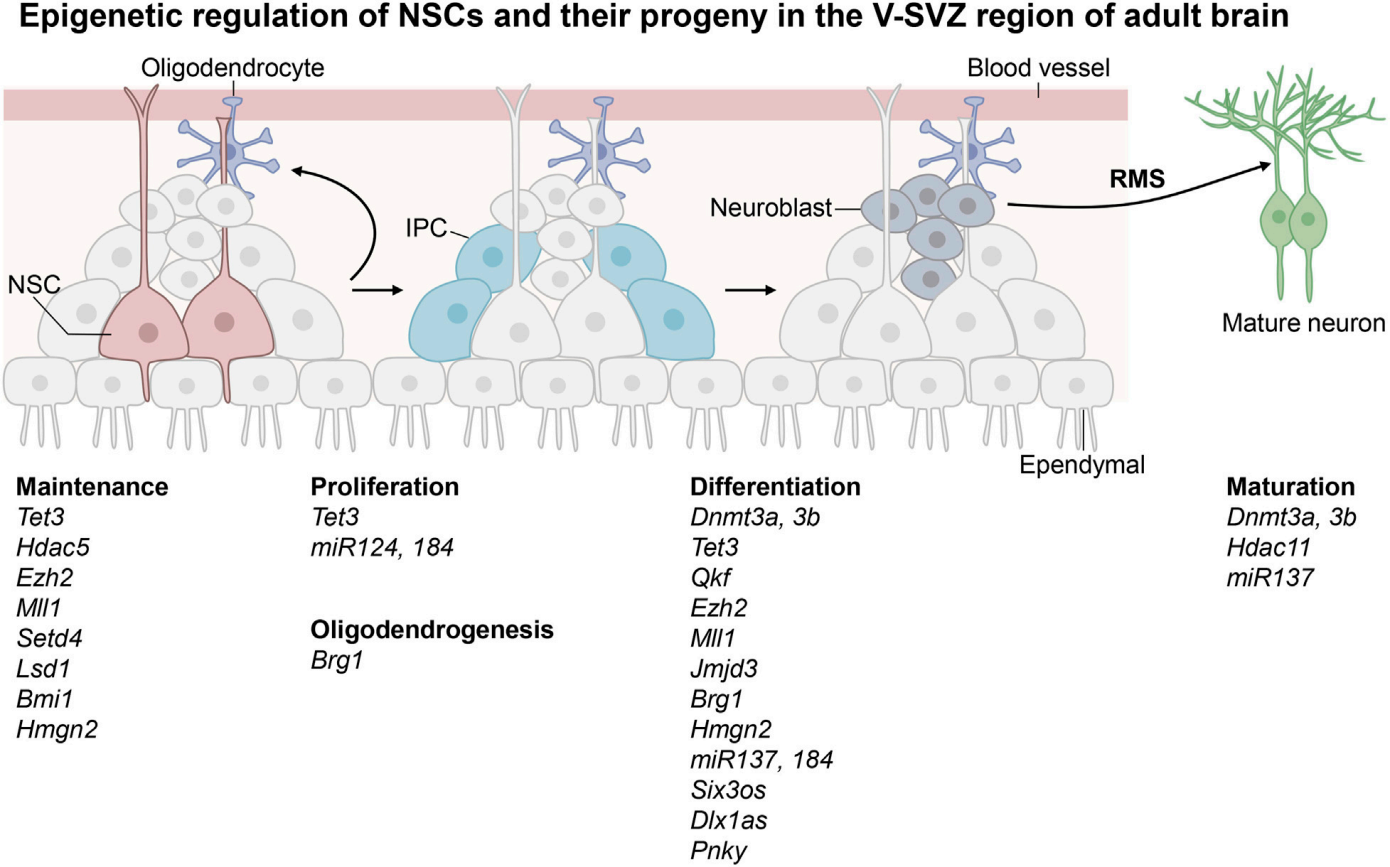
Epigenetic regulation of the neural stem cells in the ventricular-subventricular zone (V-SVZ) of adult brain. Adult neural stem cells (NSCs) in the V-SVZ give rise to intermediate progenitor cells (IPCs) before occurrence of neuroblasts. The immature neuroblasts enter rostral migratory stream (RMS) before they turn into mature neurons in the olfactory bulb. The epigenetic regulators shown in this figure are known to play important roles in maintenance, proliferation and differentiation of adult NSCs in the V-SVZ region.

**FIGURE 2:**
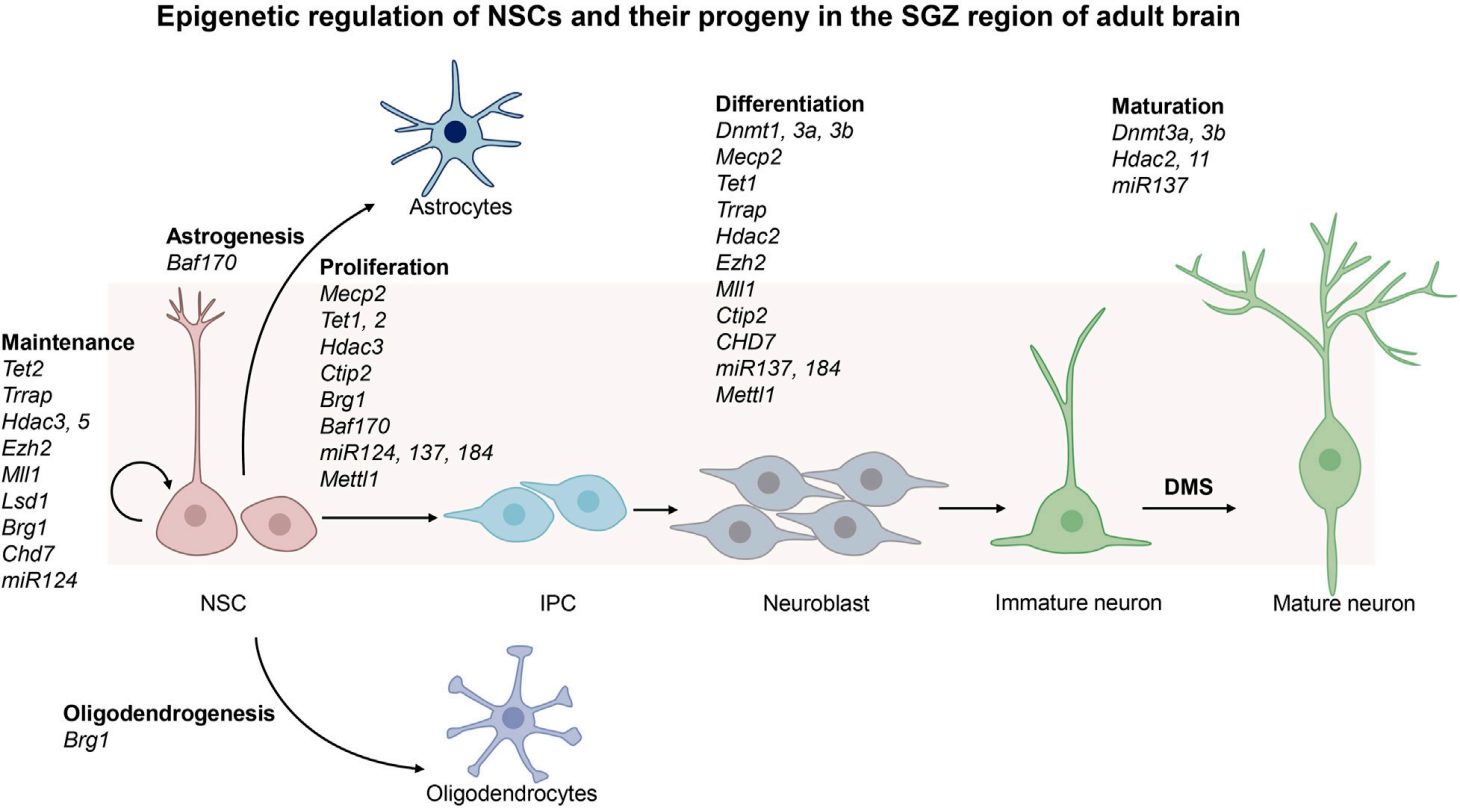
Epigenetic regulation of the neural stem cells in the subgranular zone (SGZ) of the adult brain. SGZ is located in the dentate gyrus (DG) of hippocampus. Upon activation, adult neural stem cells (NSCs) in SGZ give rise to intermediate progenitor cells (IPCs), astrocytes and oligodendrocytes. IPCs become neuroblasts, and then immature neurons before they further differentiate into mature neurons after they enter dentate migratory stream (DMS). The epigenetic regulators involved in these processes are shown in the figure.

Adult neurogenesis is a process that generates functional neurons and glial cells from adult neural stem cells (NSCs) (Ming and Song, 2011; Hsieh and Zhao, 2016; Kuhn et al., 2018; Cope and Gould, 2019; Bond et al., 2021). There are two neurogenic regions in adult mouse brain, the ventricular-subventricular zone (V-SVZ) located in the lateral ventricle and the subgranular zone (SGZ) located in the dentate gyrus (DG) of hippocampus (Bond et al., 2021; Kobayashi and Kageyama, 2021). Adult NSCs at the DG niche often adopt a radial glial morphology and thus they are also called radial glia-like neural stem cells (RGLs), whereas those at the V-SVZ niche are termed B cells (Bond et al., 2021). The adult NSCs in V-SVZ have embryonic origin and enter a quiescence state during embryonic development. They are reactivated by both intrinsic and extrinsic signals before they give rises to neurons and small populations of glial cells (Delgado et al., 2021). First, they generate intermediate progenitor cells (IPCs), which undergo further differentiation to become immature neurons called neuroblasts (Lim and Alvarez-Buylla, 2016). Neuroblasts are precursors of neural cells. They migrate through the rostral migratory stream (RMS) to the olfactory bulb (OB) where they turn into mature inhibitory interneurons that are essential for olfaction. Occasionally adult NSCs located in the V-SVZ region can also give rise to oligodendrocytes which subsequently migrate to the corpus callosum and striatum where they further differentiate into myelinated or unmyelinated oligodendrocytes. The adult NSCs in the SGZ region are located in the granular cell layer and hilus of the dentate gyrus (Vicidomini et al., 2020; Kobayashi and Kageyama, 2021). These adult NSCs are released from the quiescent state in response to neural activity and environmental factors surrounding the niche of adult NSCs. They enter dentate migratory stream (DMS) to cross the granular cell layer radially before they give rise to IPCs, which in turn become neuroblasts and undergo further differentiation in the CA3 region of the hippocampus. They are integrated into the existing neural circuits to induce plasticity with important functions in cognition such as learning and memory (Goncalves et al., 2016). A small number of NSCs in SGZ can also migrate to the hilus and granular layers of hippocampus to generate oligodendrocytes and astrocytes. In summary, adult NSCs may remain quiescent to maintain a pool of stem cells in the V-SVZ and SGZ regions. They may undergo proliferation, differentiation, migration and integration into existing neural circuits to function as mature neurons. Besides neurons, NSCs may also give rise to glial cells (Figure 1; Figure 2).

Balancing maintenance and differentiation of adult NSCs is controlled by both intrinsic and extrinsic signals. Intrinsic signals include some key transcription factors produced in the NSCs and IPCs that are important for maintenance and/or differentiation of these cells, whereas extrinsic signals generally refer to growth factors and neurotrophins secreted in the surrounding niche (Covic et al., 2010; Matsubara et al., 2021). There are a lot of studies indicating the important roles of transcription factors in NSCs and their neural development (Harada et al., 2021; Fong et al., 2022; Guo et al., 2022; Li et al., 2022; Qin et al., 2022; Fan et al., 2023). Epigenetic modifications in response to both kinds of signals are crucial for maintaining the quiescence state of NSCs and dictating their cell lineage differentiation by spatial and temporal regulation in expression of some key factors in the NSCs (Yao et al., 2016). These epigenetic regulations are mediated through DNA methylation, histone modifications, chromatin remodeling, and non-coding RNAs, etc. This review primarily focuses on epigenetic and transcriptional regulations of adult NSCs located in the V-SVZ and SGZ of the adult mouse brain, together with some referenced studies in mouse embryos or cell culture in support of the findings and conclusions in adult mice (see below).

## DNA methylation

Cytosine DNA methylation is essential for mammalian development by regulating lineage commitment in cell differentiation in embryos or stem cells (Li and Zhang, 2014; Dor and Cedar, 2018; Zeng and Chen, 2019; Chen and Zhang, 2020). It is altered in many human diseases including neurological diseases (Hamidi et al., 2015; Xie et al., 2023). It occurs primarily at the CpG sites in mammals, with S-adenyl methionine (SAM) as the donor (Zeng and Chen, 2019; Chen and Zhang, 2020). In the human and mouse brains, DNA methylation also occurs at the CpH sites (H = A, C or T), with generally much lower frequencies compared with those of CpG methylation (Guo et al., 2014).

DNA methylation is catalyzed by DNA methyltransferase (DNMT). The mammalian DNMT family mainly consists of DNMT1, DNMT3A and DNMT3B. Among them, DNMT3A and 3B are the major DNMTs for *de novo* DNA methylation, while DNMT1 is primarily involved in the maintenance DNA methylation (Chen and Zhang, 2020). DNMT1 is highly expressed in the central nervous system (CNS) of both mouse embryos and postnatal mice. It is crucial for neurogenesis as well as survival of newly generated neurons in SGZ although it does not appear to be required for the existing mature neurons (Noguchi et al., 2015; Cui and Xu, 2018). *Dnmt3a* is expressed in the NSCs of the SVZ of the mouse embryonic brain from E10.5 until E17.5, whereas *Dnmt3b* expression is detected in the ventricular zone of the brain in mouse embryos from E10.5 to E13.5 (Feng et al., 2005). Expression of both genes decreases postnatally. Nevertheless, loss of DNMT3A results in reduced number of newborn neurons in the SVZ and SGZ regions of the postnatal mouse brain (Wu et al., 2010). DNMT3A is shown to bind to the intergenic regions as well as across the transcribed regions or gene bodies of lowly expressed genes in newborn pups, in which DNA methylation occurs at the CpA sites of these regions that is required for fine-tuning of neuronal subtype-specific transcription in the adult brain (Stroud et al., 2017). DNMT3A and DNMT3B are also associated with the enhancers and gene bodies of the neuronal target genes in adult neurogenesis to establish neuron-specific methylomes and gene expression patterns that are essential for maturation and integration of newborn neurons in the adult brain (Zocher et al., 2021). They do not appear to affect proliferation or cell fate specification of newborn neurons in the adult hippocampus though. Interestingly, growth of dendrites and synaptogenesis are impaired when both DNMT3A and DNMT3B are ablated in adult NSCs, which causes learning and memory defects in the hippocampus (Zocher et al., 2021). In addition, phosphorylation of DNA methylation binding protein MeCP2 by Aurora kinase B is required for balancing proliferation and differentiation of NSCs in the adult brain through NOTCH signaling pathway (Li et al., 2014).

Ten-eleven translocation (TET) proteins are α-ketoglutarate-dependent and Fe^2+^-dependent dioxygenases that catalyze conversion of 5-methylcytosine (5 mC) to 5-hydroxymethylcytosine (5hmC), which ultimately leads to DNA demethylation (He et al., 2011; Ito et al., 2011; Wu and Zhang, 2017; Xu and Bochtler, 2020). There are three TET proteins in mammals, namely TET1, TET2 and TET3. Although it is much less abundant than 5 mC in most cell types, 5hmC is relatively enriched in the brain (Santiago et al., 2014; MacArthur and Dawlaty, 2021). TET1 may participate in SGZ neurogenesis by regulating NSC proliferation and differentiation as well as cognition in adult mice (Zhang et al., 2013). Knockdown of *Tet1* causes promoter hypermethylation in the *Dll3* (Delta-like 3) and *Notch1* genes that leads to inhibition of NOTCH signaling pathway and results in decreased NSC proliferation (Chen et al., 2021). Similarly, TET1 can also affect neurogenesis in the adult hippocampus by modulating *miR-124* expression (Choi et al., 2019). Hippocampal aging is accompanied by reduction in the TET2 protein and 5hmC levels, and *Tet2* knockdown causes impairment of neural regeneration and cognitive function. In contrast, restoration of TET2 in the mature adult hippocampus can rescue these brain defects in the hippocampus (Gontier et al., 2018). Loss of TET3 causes proliferation and differentiation defects in neural progenitor cells. Intriguingly, TET3 has been shown to maintain the stem cell pool of the V-SVZ region by binding directly to the *Snrpn* gene and represses its transcription from the paternal allele (Montalban-Loro et al., 2019).

In mammals, DNA methylation plays an important role in genomic imprinting characterized by parent-of-origin-dependent mono-allelic expression (Li, 2013; Barlow and Bartolomei, 2014; Monk et al., 2019; Tucci et al., 2019; Bartolomei et al., 2020). Genomic imprinting has been implicated in regulating dosage-sensitive gene expression in the neurogenic niche (Montalban-Loro et al., 2015; Perez et al., 2016; Lozano-Urena et al., 2017). So far, about 200 known imprinted genes have been discovered to exhibit parent-of-origin–dependent monoallelic expression patterns (Tucci et al., 2019; Xu et al., 2022). Tissue-specific imprinting has been observed for some imprinted genes such as the *Ube3a* imprinted gene at the *Snrpn* imprinted region that shows mono-allelic expression in the brain but not in the other organs (Hsiao et al., 2019; Jiang et al., 2021). It is not expressed in the embryos either. Normally, *Dlk1* is almost exclusively expressed from the paternal allele in mouse embryos, whereas it is bi-allelically expressed in the NSCs and niche astrocytes in the adult V-SVZ and SGZ regions (Ferron et al., 2011; Montalban-Loro et al., 2021). The *Dlk1* gene expresses multiple transcript isoforms encoding membrane-bound and secreted proteins of DLK1. It is reported that the secreted DLK1 by niche astrocytes interacts with its membrane-bound DLK1 present on NSCs, which is important for neurogenesis and cognition (Montalban-Loro et al., 2021). The imprinted *Igf2* gene is expressed solely from the paternal allele in the NSCs of SGZ that encodes an autocrine factor to prevent NSCs from apoptosis. In contrast, biallelic expression of *Igf2* is observed in the cerebrospinal fluid and endothelial cells of V-SVZ (Ferron et al., 2015). It is possible that dosage-sensitive transcriptional regulation of *Igf2* through its imprinted expression may be crucial for adult neurogenesis. Interestingly, IGF2 is also important for terminal differentiation of NSCs into neurons, astrocytes and oligodendrocytes through regulation of another imprinted gene *Cdkn1c* encoding a cell-cycle inhibitor p57 (Lozano-Urena et al., 2023). Thus, DNA methylation plays diverse and important roles in the maintenance, proliferation and differentiation of NSCs.

## Histone post-translational modification

Histone post-translational modifications (PTMs) refer to the covalent chemical modification on histone proteins, including acetylation, methylation, ubiquitination, phosphorylation, ribosylation, and SUMOylation, etc. (Bannister and Kouzarides, 2011; Suganuma and Workman, 2018; Talbert et al., 2019; Millan-Zambrano et al., 2022). Many transcription factors are known to be important for CNS development and adult neurogenesis. Histone PTMs facilitate recruitment of these transcription factors to their binding sites on chromatin to promote transcriptional activation or repression in the brain (Adam and Harwell, 2020; Chen and Zhang, 2020). Histone acetylation catalyzed by histone acetyltransferases (HATs) is a marker of transcriptional activation, whereas deacetylation catalyzed by histone deacetylases (HDACs) inhibits transcription. QKF, a member of the MYST family of HAT, is highly expressed in the adult V-SVZ. Its loss causes reduced neuroblast migration through RMS, and accordingly fewer interneurons can reach OB (Merson et al., 2006; Sheikh et al., 2012). TRRAP-mediated histone acetylation regulates SP1-dependent transcription in adult neurogenesis, and TRRAP deletion inhibits self-renewal and differentiation potentials of adult NSCs (Yin et al., 2023). Furthermore, activation of adult NSCs is shown to be dependent on regulation of histone acetylation via ATP-citrate lyase (Liu et al., 2023b). In contrast, HDACs remove acetyl groups from histones to repress transcription (Chen and Zhang, 2020; Li et al., 2020). There are eighteen known HDACs that are classified into four groups (Class I, Class II, Class III and Class IV). HDAC2 and HDAC3 belong to class I HDACs. HDAC2 is shown to be required for transcriptional silencing during neuronal differentiation in adult neurogenesis, and its loss results in death of neurons at the maturation stages during adult neurogenesis (Jawerka et al., 2010). HDAC3 regulates proliferation and cell cycle progression in adult neurogenesis mainly through acetylation of G2/M cyclin-dependent kinase 1 (CDK1) (Jiang and Hsieh, 2014). HDAC5 belongs to class II HDACs and it is reported to interact with the orphan nuclear receptor TLX, a transcription factor necessary for NSC proliferation and self-renewal in cell culture (Sun et al., 2007). This interaction causes transcriptional repression of some TLX target genes in cell cycle regulation, including the cyclin-dependent kinase inhibitor *p21* and the tumor suppressor gene *pten* in the NSC cell culture experiments (Sun et al., 2007). Class III HDACs consist of sirtuins such as SIRT1 and SIRT5 that are required for cell fate determination of adult NSCs and maintenance of the nervous system during aging, although the specific mechanism is still unclear (Rafalski and Brunet, 2011; Herskovits and Guarente, 2014; Santos et al., 2021). HDAC11 is the only member of Class IV HDAC family that is currently known to act in neuronal maturation by regulating dendritic length and complexity (Nunez-Alvarez and Suelves, 2022).

Histone methylation modification is catalyzed by histone methyltransferases (HMTs), with methyl group added onto the lysine (K) and arginine (R) residues. These may be either transcriptional activation marks or repression marks (Bedford and Clarke, 2009; Guccione and Richard, 2019; Jambhekar et al., 2019; Morgan and Shilatifard, 2020). Polycomb (PcG) proteins are important for formation of some repressive chromatin marks. There are two types of PcG complexes: polycomb repressive complex 1 (PRC1) and polycomb repressive complex 2 (PRC2) (Almeida et al., 2020; Blackledge and Klose, 2021). Both PRC complexes regulate chromatin accessibility and expression of region-specific transcription factors in NPC differentiation and brain regionalization (Eto and Kishi, 2021). EZH2 is a key member of PRC2 that catalyzes H3K27me3 formation. It is expressed in the V-SVZ and SGZ of adult mice, and loss of EZH2 in these regions affects both differentiation and maintenance of neural stem cells and progenitor cells (Rhodes et al., 2018). MLL1 is an HMT of the Trithorax (TrxG) complex, and it is required for neurogenesis in V-SVZ by regulating transcription factor DLX2 (Lim et al., 2009). MLL1-deficient NSCs in SGZ can survive and proliferate. And they can differentiate into glial cell lineages, but their neuronal differentiation potential is severely impaired. MLL1 is also required for maintaining NSC positional identity through regulation of region-specific transcription factors such as NKX2-1 (Delgado et al., 2020). Without MLL1, expression of some dorsal identity genes increases in the neurons derived from adult NSCs. SETD4, an H4K20me3 writer, maintains the NSC population in the adult brain, and loss of SETD4 leads to depletion of adult NSCs in the mutant mice (Cai et al., 2022). In contrast, lysine-specific demethylase 1 (LSD1) that catalyzes histone lysine demethylation is required for NSC proliferation. It is recruited by TLX to suppress expression of its target genes after removal of activation methylation marks (Sun et al., 2010). JMJD3, a histone H3 lysine 27 (H3K27) demethylase, regulates the *Dlx2* enhancer during NSC differentiation (Park et al., 2014).

Other histone modifications also play crucial roles in neurogenesis. BMI1 that is a member of the PcG family proteins and a component of polycomb repressive complex 1 (PRC1), cooperates with RING1, another component of PRC1, in ubiquitination of K119 of H2A. Loss of *Bmi1* affects self-renewal of NSCs in the SVZ of adult mice partly through deregulation of cell cycle inhibitors such as p16^Ink4a^ and P19^Arf^ (Pardal et al., 2005). There are some well-documented studies regarding the important functions of other components of PRC1 such as RYBP, YAF2, PCGF5 and PCGF6 in neural differentiation from mouse embryonic stem cells (ESCs) or from human induced pluripotent stem cells (iPSCs) (Yao et al., 2018; Lan et al., 2022; Liu et al., 2023a). It is worth noting that RING1 (also called PCGF1) is required for neuronal subtype specification in the enteric nervous system of adult mouse (Putra et al., 2023). Nevertheless, it awaits further investigation if they may play similar roles in adult NSCs.

Taken together, histone modifications catalyzed by different enzymes are important for adult neurogenesis that can either activate or repress key target genes required for proper neurogenesis.

## Chromatin remodeling

Chromatin-remodeling complexes contain ATPases to modulate chromatin structure and gene expression (Becker and Workman, 2013; Hota and Bruneau, 2016; Ahmad et al., 2022). In mammals, they can be divided into four major subfamilies based on the characteristics of their ATPase catalytic domains: BAF (SWI/SNF), ISWI, CHD/NuRD and INO80/SWR (Hota and Bruneau, 2016). *Bcl11b*/*Ctip2* encodes a subunit of BAF regulating survival, differentiation as well as circuit integration of the granule neurons generated from SGZ. Loss of *Ctip2* expression in the adult hippocampus and dentate gyrus results in reduced proliferation and differentiation of NSCs (Simon et al., 2012; Simon et al., 2016). Deletion of BRG1 in the BAF family, which directly interacts with the transcription factor PAX6, causes differentiation of adult NSCs into ependymal lineages in the V-SVZ, whereas migrating neuroblasts become glial cells in the RMS during their migration to OB (Ninkovic et al., 2013). BRG1 is also involved in the maintenance and proliferation of hippocampal progenitor cells through regulation of the p53-p21 axis (Petrik et al., 2015). Loss of BAF170, another subunit of the BAF complex, results in premature differentiation of NSCs into astrocytes in the SGZ that causes depletion of NSCs (Tuoc et al., 2017). CHD7, a member of the CHD family, maintains the NSC quiescent status in the hippocampal SGZ through induced expression of *Hes5*, a target gene of NOTCH signaling (Jones et al., 2015). It is required for expression of SOX4 and SOX11, two transcription factors necessary for neuronal differentiation (Feng et al., 2013). Taken together, chromatin-remodeling complexes are required for neurogenesis by modulating chromatin structure and gene expression.

## Nucleosome and chromatin organization

Nucleosome positioning affects transcription. It is also important for neural development. Indeed, high-mobility group nucleosomal binding domain 2 (HMGN2) was shown to be expressed in the SVZ and SGZ regions of adult mouse brain and loss of HMGN2 caused reduced self-renewal and increased differentiation of adult NSCs, which resulted in microcephaly (Gao et al., 2020). There was correlation between nucleosome occupancy and histone modifications in the genome, and there was also an increase in average length of the nucleosomes in the differentiated neuronal cells derived from mouse ESCs (Teif et al., 2012). Nucleosome positioning may be also important for neural differentiation from human iPSCs, with an increase in the number of positioned nucleosomes as well as repositioning of nucleosomes upon differentiation of human iPSCs (Harwood et al., 2019). CTCF is an important regulator in enhancer-promoter interactions and 3D genome organization necessary for proper gene expression (Ghirlando and Felsenfeld, 2016; Chen and Long, 2023). It is required for embryonic neural development and neural differentiation from mouse and human ESCs (Bonev et al., 2017; Modrek et al., 2017; Arzate-Mejia et al., 2018; Pekowska et al., 2018; Kubo et al., 2021; Dong et al., 2022). However, it remains to be tested if similar nucleosome positioning and CTCF-mediated 3D chromatin organization effects may be observed in the process of neural differentiation of the adult NSCs.

## Non-coding RNAs

Non-coding RNA (ncRNA) does not appear to encode any long peptide in its sequence (Chen and Rechavi, 2022; Nojima and Proudfoot, 2022; van Zonneveld et al., 2023). However, some ncRNAs have been shown to display important biological functions in many cellular processes including neural development (Soutschek and Schratt, 2023). Huge numbers of ncRNAs have been discovered and millions of ncRNAs may exist in the mammalian genome. There are a few known kinds of ncRNAs such as microRNAs (miRNAs) and long non-coding RNAs (lncRNAs).

One of the first discovered ncRNAs is miRNA that contains a small single-stranded RNA molecule with approximately 22 nucleotides in length (Shang et al., 2023). More than one thousand miRNAs have been discovered in mouse or humans. In mammals miRNA usually targets the 3′UTR of mRNAs with imperfect complementary to cause translational inhibition, whereas it may trigger transcriptional repression or RNA degradation with nearly perfect complementation to its targets in plants (Chen and Rechavi, 2022). *miR124* is one of highly abundant miRNAs present in the brain (Sun et al., 2013). It targets *Sox9,* a key transcription factor in neural development, to regulate neural regeneration in the V-SVZ region during the transition from IPCs to neuroblasts (Cheng et al., 2009). EZH2 is targeted by *miR137*, another miRNA, to regulate NSC proliferation and differentiation which is under the control of MeCP2 and SOX2 (Szulwach et al., 2010). It has been shown that *miR137* plays a role in neuronal maturation and dendritic morphogenesis by targeting MIB1 in the ubiquitin-regulated pathway (Smrt et al., 2010). Another miRNA *miR184* which is regulated by MBD1 promotes proliferation but inhibits differentiation of NSCs (Liu et al., 2010). A cluster of six miRNAs have been shown to be required for NSC proliferation at the expense of oligodendrocytes (Favaloro et al., 2022).

Generally, lncRNAs are more than 200 nucleotides in length, with no obviously translated protein product (Yao et al., 2019; Andergassen and Rinn, 2022; Nojima and Proudfoot, 2022; Mattick et al., 2023). There are hundreds of thousands of lncRNAs in mammals. The imprinted *H19* gene product is the first discovered lncRNA (Brannan et al., 1990; Bartolomei et al., 1991). *Xist* involved in mammalian X chromosome inactivation is another well-known founding member of lncRNAs (Loda et al., 2022). LncRNAs may regulate gene expression in a tissue-specific pattern (Ernst and Morton, 2013; Zhang et al., 2019; Statello et al., 2021). They are prevalently expressed in the brain that contains the highest number of tissue-specific lncRNAs (Francescatto et al., 2014; Washietl et al., 2014; Ninou et al., 2021). Depletion of two lncRNAs, *Six3os* and *Dlx1as,* in the NPCs of the adult V-SVZ region results in increased astrocyte differentiation at the expense of neurons (Ramos et al., 2013). The lncRNA *Pnky* interacts with PTBP1 to regulate neural differentiation from NSCs *in vivo*, and *Pnky* knockdown increases neural commitment in differentiation of NSCs (Ramos et al., 2015). However, further studies are needed to elucidate the molecular mechanisms of lncRNAs in transcriptional regulation of adult NSCs.

## RNA methylation

There are already more than 100 different kinds of known RNA modifications. RNA methylation is among the most common RNA modifications that may play important roles in neural development (Yoon et al., 2018). Without an rRNA methyltransferase FBL, neural differentiation and neuronal progression from NSCs is inhibited in mouse embryos because there is reduced translation of EZH2 and KDM6b (Wu et al., 2022). YTHDF2, an m6A reader, is required for self-renewal and neural differentiation of embryonic NSCs in mouse through RNA degradation (Li et al., 2018). It has also been shown that FMRP is a reader for m6A modified mRNAs and promotes their nuclear export in order to fulfill their roles in cell cycle progression and maintenance of neural progenitors derived from NSCs in mouse embryos (Edens et al., 2019). These findings may need to be tested and confirmed in adult NSCs. Interestingly, ablation of METTL1 inhibits m7G RNA methylation and causes reduced hippocampal neurogenesis from NSCs in adult mice (Li et al., 2023). Therefore, at least some RNA methylation seems to be important for adult NSCs.

## Perspectives

The stem cell state of adult NSCs is maintained by both intrinsic factors and extrinsic signals. Through spatial and temporal regulation of expression of key transcription factors and signaling pathway modulators, many epigenetic regulators have already been shown to be required for maintenance of NSCs in the adult brain. They also play important roles in balancing proliferation and differentiation of NSCs in adult neural development. Interestingly, the epigenetic modifications established during early development may exert significant influence on neurogenesis in the adult brain. Consistent with this, adult NSCs are thought to be derived from embryonic radial glial (RG) cells and reversibly enter the quiescent state after they exit cell cycle. This also implies that the impact of epigenetic modifications established during early development may need to be taken into account in analyses of the mechanisms underlying maintenance, proliferation and differentiation of adult NSCs.

DNA and RNA methylation, histone modifications, chromatin remodeling, nucleosome positioning, 3D chromatin organization as well as non-coding RNAs may function in distinct pathways to ensure adult neurogenesis to progress in an orderly fashion. As shown in other cells and model systems, these epigenetic modifications do not act alone and may indeed function synergistically in order for adult NSCs to attain various cellular states. The cross-talk among different epigenetic modifications may be important for integration of intrinsic and extrinsic factors in cell fate transition of adult NSCs. Furthermore, there are a lot more epigenetic modifications that have yet to be discovered, and undoubtably some of them may be involved in regulation of adult NSCs. It remains to be explored how other new epigenetic modifications may modulate adult NSCs. It is also important to examine how many transcription factors and epigenetic regulators may share their functions in the adult NSCs in the V-SVZ and SGZ regions (Figure 1; Figure 2). It remains to be tested if these findings may be applicable to adult NSCs in human brains.

Despite much progresses in adult NSCs over the last few decades, it is not that clear how adult NSCs maintain their quiescent cellular state and how external signals and intrinsic factors drive them to re-enter cell cycle and give rise to different cell lineages before integration into the neural circuits. Application of high-throughput technologies in epigenetic research such as RNA-seq, WGBS, ChIP-seq and ATAC-seq, in combination with single cell analyses, will help us to better understand dynamic transcriptional regulation of adult NSCs in their cell fate transition and specification. It is still in the infancy stage to uncover epigenetic regulators and modifications in adult NSCs and their roles in adult neurogenesis and neural plasticity.
